# Functional Magnetic Resonance Urography in Ureteropelvic Junction Obstruction: Proposal for a Pediatric Quantitative Score

**DOI:** 10.3389/fped.2022.882892

**Published:** 2022-06-16

**Authors:** Maria Beatrice Damasio, Fiammetta Sertorio, Michela Cing Yu Wong, Irene Campo, Marcello Carlucci, Luca Basso, Lorenzo Anfigeno, Monica Bodria, Angela Pistorio, Giorgio Piaggio, Gian Marco Ghiggeri, Girolamo Mattioli

**Affiliations:** ^1^Radiology Department, Istituto di Ricovero e Cura a Carattere Scientifico (IRCCS) Istituto Giannina Gaslini, Genoa, Italy; ^2^Pediatric Surgery Department, Istituto di Ricovero e Cura a Carattere Scientifico (IRCCS) Istituto Giannina Gaslini, Genoa, Italy; ^3^Radiology Department, Ospedale di Conigliano – Unità Locale Socio-Sanitaria (ULSS) 2 Marca Trevigiana, Conegliano, Italy; ^4^Department of Health Sciences, Radiology Department, University of Genoa, Genoa, Italy; ^5^Nephrology and Renal Transplantation Department, Istituto di Ricovero e Cura a Carattere Scientifico (IRCCS) Istituto Giannina Gaslini, Genoa, Italy; ^6^Epidemiology and Biostatistics Department, Istituto di Ricovero e Cura a Carattere Scientifico (IRCCS) Istituto Giannina Gaslini, Genoa, Italy

**Keywords:** kidney, fMRU, CAKUT, UPJ, score

## Abstract

**Background:**

Ureteropelvic junction obstruction (UPJO) is one of the most frequent causes of congenital hydronephrosis. It is essential to distinguish UPJO which needs surgical treatment. fMRU combines high quality morphological details of the kidney and excretory pathways with functional data.

**Objective:**

This study aims to introduce a new radiological score based on fMRU findings to be able to differentiate surgical from non-surgical kidneys.

**Materials and Methods:**

We retrospectively selected patients with hydronephrosis due to UPJO who underwent fMRU (January 2009–June 2018). A multidisciplinary team identified a list of fMRU morpho-functional predictive variables to be included in the analysis. To evaluate the role of different independent variables in predicting the outcome, a multivariable logistic regression model has been performed; the outcome variable was the surgical intervention. For each predictive variable, Odds Ratio and 95% Confidence Intervals were calculated. The likelihood ratio test was used to assess the significance of the variables. Using the regression model, we assigned a numerical value to each predictive variable, rounding up the beta-coefficients. The cut-off value of the total score was obtained from the ROC curve analysis.

**Results:**

A total of 192 patients were enrolled, corresponding to 200 pathological kidneys. All of them underwent fMRU; 135 were surgically treated, while 65 underwent ultrasound or MRU follow-up. Predictive variables significantly associated with surgery resulted to be the urographic phase, the presence of abnormal vessels, and a baseline anterior-posterior pelvic diameter >23 mm. Beta coefficients of the logistic regression model were then converted in scores. The ROC curve of the score showed high sensitivity (84.3%) and specificity (81.3%) with a cut-off > 2.5.

**Conclusions:**

We propose a new fMRU score able to identify surgical vs. non-surgical kidneys with UPJO.

## Introduction

Congenital hydronephrosis is diagnosed in 3–5% of all prenatal ultrasounds (US) (1). While most hydronephrosis are resolved spontaneously, both mild and severe forms may evolve to end stage renal disease (2–4).

Ureteropelvic junction obstruction (UPJO) (1:1,500 newborns) is a congenital anomaly associated with hydronephrosis that can be corrected through surgery (5, 6).

UPJOs are classified as intrinsic or extrinsic causes. Intrinsic forms usually arise from an adynamic ureteral segment or cicatricial post-operative factors whereas extrinsic types are characterized by aberrant crossing vessel (CV), kinkings or adhesions over the ureteropelvic junction (UPJ). UPJOs may result from both intrinsic and extrinsic obstruction (7).

It is essential to distinguish between forms that may be resolved spontaneously from those that require surgical treatment to prevent further loss of renal function (8).

International guidelines (8) are based on radiological data, acquired through techniques such as US and Dynamic Renal Scintigraphy (DRS). US is a diagnostic imaging technique used to evaluate hydronephrosis while DRS is considered the gold standard technique to study the obstruction and the Split Renal Function (SRF) between the two kidneys (9, 10).

The use of functional Urographic Magnetic Resonance Imaging (fMRU) has progressively increased in the assessment of Congenital Anomalies of Kidneys and Urinary Tract (CAKUT) (11–16). fMRU can define the structure of the excretory pathway and the presence of several anomalies while simultaneously it can quantify renal function through SRF and renogram curves (17). In a recent study, fMRU showed a good reliability both for anatomical and functional evaluation (18). To our knowledge, fMRU has never been applied as a grading tool for obstructive uropathies while DRS is considered the gold standard for CAKUT (19).

Therefore, the aim of this study is to introduce a fMRU score to identify patients with UPJO who require surgical correction based on quantitative data. The study compares fMRU results to surgical findings and outcomes in a single pediatric center over 10 years.

## Materials and Methods

Between January 2009 and June 2018, we retrospectively analyzed all patients who underwent fMRU for hydronephrosis, diagnosed through renal US (anterior-posterior pelvic diameter, APD ≥ 1.5 cm) (20). Patients with associated renal phenotype like megaureter or homolateral double collecting system were excluded.

fMRU was performed in all patients using a standardized Institute protocol, following the previous one published by Vivier et al., and data was analyzed using the available ImageJ MRU software (12, 13). The parameters considered for fMRU score were affected side, pelvic morphology and rotation, APD pre and post-Furosemide administration, delta APD (difference between APD before and after diuretic), presence/absence and type of abnormal vessel, presence/absence of basal dilated ureter and calicectasia, renographic curve, and SRF evaluated both with AUC and Patlak methods. Pelvic morphology was classified into normal, extra-renal, intra-renal, plongeant, pelvic agenesia (direct connection between calyxes and ureter) while pelvic rotation was classified into normal, anterior, posterior, and pelvic agenesia. APD was measured on transversal image at the maximal diameter of intrarenal pelvis (21). Renographic curve is a time-intensity curve calculated by drawing a region of interest (ROI) on Gradient Echo T1-weighted coronal post-contrast images encompassing the entire kidney, including parenchyma and the collecting system (13). Blind evaluation of fMRU images was carried out by an expert pediatric radiologist. fMRU results were compared with surgical data, considered the gold standard to define the etiology of UPJO.

According to current guidelines, indications for surgical intervention are: impaired SRF <40%, a decrease of SRF >10% in subsequent studies, poor drainage function after Furosemide, increased APD on US, and grade III and IV dilation as defined by the Society for Fetal Urology (8, 22).

According to our institution's guidelines, the presence of an obstructing CV or obstructive symptoms (recurrent flank pain, urinary tract infections) are also considered indications for surgery.

All the resected UPJ were analyzed by a pathologist.

Follow-up was performed by fMRU or US in both groups (surgical and non-surgical patients). The parameters taken into consideration during the follow-up were: APD (variation >5 mm), calicectasia (grade and extension) and renal parenchymal thickness. Based on the fMRU and/or sonographic appearance of these features the pediatric radiologist defined three categories for clinical outcomes: improved, stable, or worsened.

As the study was observational and retrospective in design and did not examine patients' personal information, ethical committee approval was not required.

### Statistical Methods

Firstly, descriptive statistics were performed, categorical variables were reported in terms of absolute frequencies and percentages while quantitative variables were reported in terms of mean values with SD or in terms of median values and first and third quartiles (1st−3rd q). Statistical analysis of frequencies was carried out using the chi square test or Fisher's Exact test (if expected frequency was <5). Comparison of quantitative variables between 2 groups (patients with/without surgical intervention) was done by means of the non-parametric Mann-Whitney *U*-test as data were not normally distributed. Normality of variables was tested by means of the Shapiro-Wilk test.

Finally, to evaluate the role of different independent variables in predicting the outcome, a multivariable logistic regression model has been performed; the outcome variable was the surgical intervention (yes, coded “1”/no, coded “0”). Clinically relevant or statistically significant variables evaluated at bivariate analysis were included in the model. Some quantitative variables (*example*: age at first MRI, SRF AUC, DAP pre-Furosemide administration, antero-posterior diameter evaluated at baseline) were dichotomized based on the best cut-off value obtained by means the ROC curve method. This method allows to obtain the best cut-off value that can discriminate between subjects who had to be operated or not (23).

Some quantitative variables were not included into the logistic regression model as they were auto-correlated (*example*: Split Renal Function “Patlak” and Split Renal Function AUC); the correlation has been evaluated by means of the Spearman's Rank order coefficient (r_S_).

The odds ratios (ORs) with their 95% CI have been calculated and reported. The log-likelihood ratio test (LR test) has been used for testing variables statistical significance. The backward approach (that consists in removing non-significant variables from the saturated model) has been used for evaluating the model. The area under the ROC curve of the model has been used as an indicator of goodness of fit.

Rounding up the regression coefficient (beta) of the statistically significant variables in the logistic regression model to the nearest *x* 0.5 value or to the nearest integer, a score has been calculated for each significant variable. All these scores were summed up to obtain for each patient a “total prediction score”. Finally, the best cut-off that could discriminate between patients who should undergo surgery or not has been calculated by means of the ROC curve method.

All the statistical tests were two-sided and a *P*-value < 0.05 was considered statistically significant. Statistica” (release 9.1, StatSoft Corporation, Tulsa, OK, USA) was used for all the bivariate analyses; MedCalc was used for the ROC curve analysis; “Stata” (release 7.0, College Station, TX, USA) was used for the Fisher's exact test, for the Cohen's *k* and for the multivariable logistic regression model.

## Results

According to our inclusion and exclusion criteria, 192 patients (61% males and 39% females) were enrolled in the study, corresponding to 200 pathological kidneys, with a median age at fMRU of 5.1 years (1.2–11.2, 1°-3° quartile). The affected side was the left one in 124 and the right one in 76 cases with a predominance (62%) of the left side. The anatomical features of pathological kidneys are shown in Table 1.

**Table 1:** Anatomical features of the kidneys included in the study (*n* = 200).

	***N* (%)**
**Affected kidney:**	
Right	76 (38.0%)
Left	124 (62.0%)
**Pelvic morphology:**	
Normal	172 (86.0%)
Extra-renal	15 (7.5%)
Intra-renal	10 (5.0%)
Plongeant	2 (1.0%)
Pelvic agenesia	1 (0.5%)
**Pelvic rotation:**	
Normal	145 (72.5%)
Anterior	24 (12.0%)
Posterior	30 (15.0%)
Pelvic agenesia	1 (0.5%)
**Abnormal vessel:**	
None	123 (61.5%)
Yes, non-obstructive	29 (14.5%)
Yes, obstructive	48 (24.0%)
**MRI diagnosis:**	
Intrinsic UPJ obstruction (UPJ-IO)	122 (61.0%)
Intrinsic cicatricial UPJ defect (UPJ-IC)	25 (12.5%)
Extrinsic vascular UPJ defect (UPJ-EV)	50 (25.0%)
Extrinsic adherential UPJ defect (UPJ-EA)	3 (1.5%)
**Type of abnormal vessel (*****n*** **=** **77):**	
Polar artery	64 (83.1%)
Secondary branch of renal artery	10 (13.0%)
Secondary branch of renal vein	3 (3.9%)

*UPJ, Uretero-Pelvic Junction. Plongeant: downward development*.

After fMRU, 135/200 kidneys underwent surgery while 65/200 kidneys underwent instrumental follow-up (US or fMRU). In one case the UPJ stenosis was not confirmed by intraoperative ascending pyelography.

In 6/192 patients, Gadolinium-based contrast medium (Gadoteric Acid) was not administered due to counter-indications. In 31/192 patients (25 surgical and 6 non-surgical) it was not possible to perform the functional phase, while in 1/192 cases the urographic phase was not performed due to early interruption of the examination.

The median age at surgery was 5.1 (1st−3rd q: 1.4–11.4) years with a median interval between fMRU and surgery of 2 months. Patients who underwent surgery had a mean follow-up of 25 months (SD: 24 months), while non-surgical patients had a mean follow-up of 30 months (SD: 26 months) from first fMRU.

All the resected UPJ were analyzed by pathologist who confirmed the diagnosis.

At a bivariate analysis (Table 2) the morphological parameter with the greatest statistical significance between the two subgroups (“surgical kidney” and “non-surgical kidney”) turned out to be the presence of the abnormal vessel obstructing the UPJ (93.8%) regardless of the type of vessel. Moreover, the dilation of the calyxes and the renal pelvis, both in basal conditions and after Furosemide administration results were statistically significant. The remaining parameters such as the morphology and rotation of the pelvis and the dilation of the ureter were not significantly different between the two groups.

**Table 2:** Comparison between operated (*n* = 135) and non-operated kidneys (*n* = 65) and fMRU parameters.

	**Surgical intervention**		***P***
	**Yes, *n*/*N* (%)**	**No, *n*/*N* (%)**	
**Kidney of:**			
Male patient	80/123 (65%)	43/123 (35%)	0.35^#^
Female patient	55/77 (71.4%)	22/77 (28.6%)	
**Kidney with previous surgical intervention [*****n*** **=** **173]:**			
Yes	28/43 (65.1%)	15/43 (34.9%)	0.43^#^
No	93/130 (71.5%)	37/130 (28.5%)	
**Kidney:**			
Right	58/76 (76.3%)	18/76 (23.7%)	0.037^#^
Left	77/124 (62.1%)	47/124 (37.9%)	
**Date of MRI:**			
2009–2013	63/97 (64.9%)	34/97 (35.1%)	0.45^#^
2014–2018	72/103 (69.9%)	31/103 (30.1%)	
**MRI diagnosis:**			
Intrinsic UPJ obstruction (UPJ-IO)	73/122 (59.8%)	49/122 (40.2%)	0.0001^§^
Intrinsic cicatricial UPJ defect (UPJ-IC)	13/25 (52.0%)	12/25 (48.0%)	
Extrinsic vascular UPJ defect (UPJ-EV)	46/50 (92.0%)	4/50 (8.0%)	
Extrinsic adherential UPJ defect (UPJ-EA)	3/3 (100%)	0/3 (0.0%)	
**Pelvic morphology:**			
Normal	115/172 (66.9%)	57/172 (33.1%)	0.96^§^
Extra-renal	11/15 (73.3%)	4/15 (26.7%)	
Intra-renal	7/10 (70%)	3/10 (30%)	
Plongeant	1/2 (50%)	1/2 (50%)	
Pelvic agenesia	1/1 (100%)	0/1 (0%)	
**Pelvic rotation:**			
Normal	96/145 (66.2%)	49/145 (33.8%)	0.65^§^
Anterior	15/24 (62.5%)	9/24 (37.5%)	
Posterior	23/30 (76.7%)	7/30 (23.3%)	
Pelvic agenesia	1/1 (100%)	0/1 (0%)	
**Abnormal vessel:**			
None	78/123 (63.4%)	45/123 (36.6%)	<0.0001^#^
Yes, non-obstructive	12/29 (41.4%)	17/29 (58.6%)	
Yes, obstructive	45/48 (93.8%)	3/48 (6.3%)	
**Type of abnormal vessel [*****n*** **=** **77]:**			
Polar artery	45/64 (70.3%)	19/64 (29.7%)	0.08^§^
Secondary branch of renal artery	10/10 (100%)	0/10 (0%)	
Secondary branch of renal vein	2/3 (66.7%)	1/3 (33.3%)	
**Basal dilated ureter:**			
No	103/147 (70.1%)	44/147 (29.9%)	0.20^#^
Yes	32/53 (60.4%)	21/53 (39.6%)	
**Calicectasis:**			
Yes pre- and post-Furosemide	128/176 (72.7%)	48/176 (27.3%)	0.0001^§^
No pre-/Yes post-Furosemide	4/11 (36.4%)	7/11 (63.6%)	
No pre- and post-Furosemide	3/13 (23.1%)	10/13 (76.9%)	
APD Pre-Furosemide, median (1st−3rd q)	28 (18.5–37) [*n* = 135]	15 (13–20) [*n* = 65]	<0.0001^**∧**^
APD Post-Furosemide, median (1st−3rd q)	33 (24–44) [*n* = 127]	23 (18–28) [*n* = 65]	<0.0001^**∧**^
Delta% APD [(Post *minus* pre-Furosemide)/Pre] × 100, Median (1st−3rd q)	14.0 (0.0–38.9) [*n* = 127]	35 (18.2–64.3) [*n* = 65]	0.0007^**∧**^
Age at 1st MRI ≤ 6.93	85/118 (72%)	33/118 (28%)	0.10^#^
>6.93	50/82 (61%)	32/82 (39%)	
SRF AUC ≤ 38	63/75 (84%)	12/75 (16%)	<0.0001^#^
>38	47/94 (50%)	47/94 (50%)	
APD pre-Furosemide ≤ 23	47/100 (47%)	53/100 (53%)	<0.0001^#^
>23	88/100 (88%)	12/100 (12%)	
**Urographic phase:**			
Ready complete	10/43 (23.3%)	33/43 (76.7%)	<0.0001^#^
Delayed complete	47/73 (64.4%)	26/73 (35.6%)	
Delayed incomplete	51/54 (94.4%)	3/54 (5.6%)	
Absent	19/21 (90.5%)	2/21 (9.5%)	
**Renographic curve:**			
Within normal limits	10/38 (26.3%)	28/38 (73.7%)	<0.0001^#^
Borderline	46/70 (65.7%)	24/70 (34.3%)	
Accumulating	55/63 (87.3%)	8/63 (12.7%)	

*UPJ, Uretero-Pelvic Junction; APD, anterior-posterior diameter in millimeters. Plongeant: downward development*.

*Figures in round parentheses represent row percentages*.

*^#^P: Pearson's Chi-square test*.

*^§^P: Fisher's Exact test*.

*^**∧**^P: Mann-Whitney U-test*.

Finally, to evaluate the role of different independent variables in predicting the surgical intervention, a multivariable logistic regression model was performed. As shown in Table 3, there were three predictors of surgical intervention: the urographic phase, the presence of abnormal vessels, and a value of pre-Furosemide administration APD >23 mm.

**Table 3:** Multivariable logistic regression model (*N* = 168); outcome variable: surgical intervention: yes (109/168; 64.9%).

**Predictive variables**	**OR**	**IC 95%**	**^**##**^ *P***	**Beta**	**Score**
**Urographic phase: (** ***Reference category: ready complete)***					
Delayed complete	6.05	(1.93–18.93)	<0.0001	1.800	2
Delayed incomplete	46.85	(9.51–230.75)		3.847	4
Absent	54.14	(5.27–556.41)		3.992	4
**Abnormal vessels: (** ***Reference category: absent*** **)**					
Present, without conflict	0.69	(0.2–2.35)	0.0002	0.366	0.5
Present, with conflict	10.47	(2.56–42.86)		2.349	2.5
DAP pre-Furosemide > 23 mm (*reference ≤ 23 mm)*	8.99	(3.31–24.39)	<0.0001	2.196	2
Area of the ROC curve of the model: 0.904					

^##^*P: Test “Likelihood Ratio” (LR test)*.

Beta coefficients of the logistic regression model were then converted in scores (Table 3), rounding up the values to the nearest integer or to the x 0.5 decimal value.

**Figure 1:**
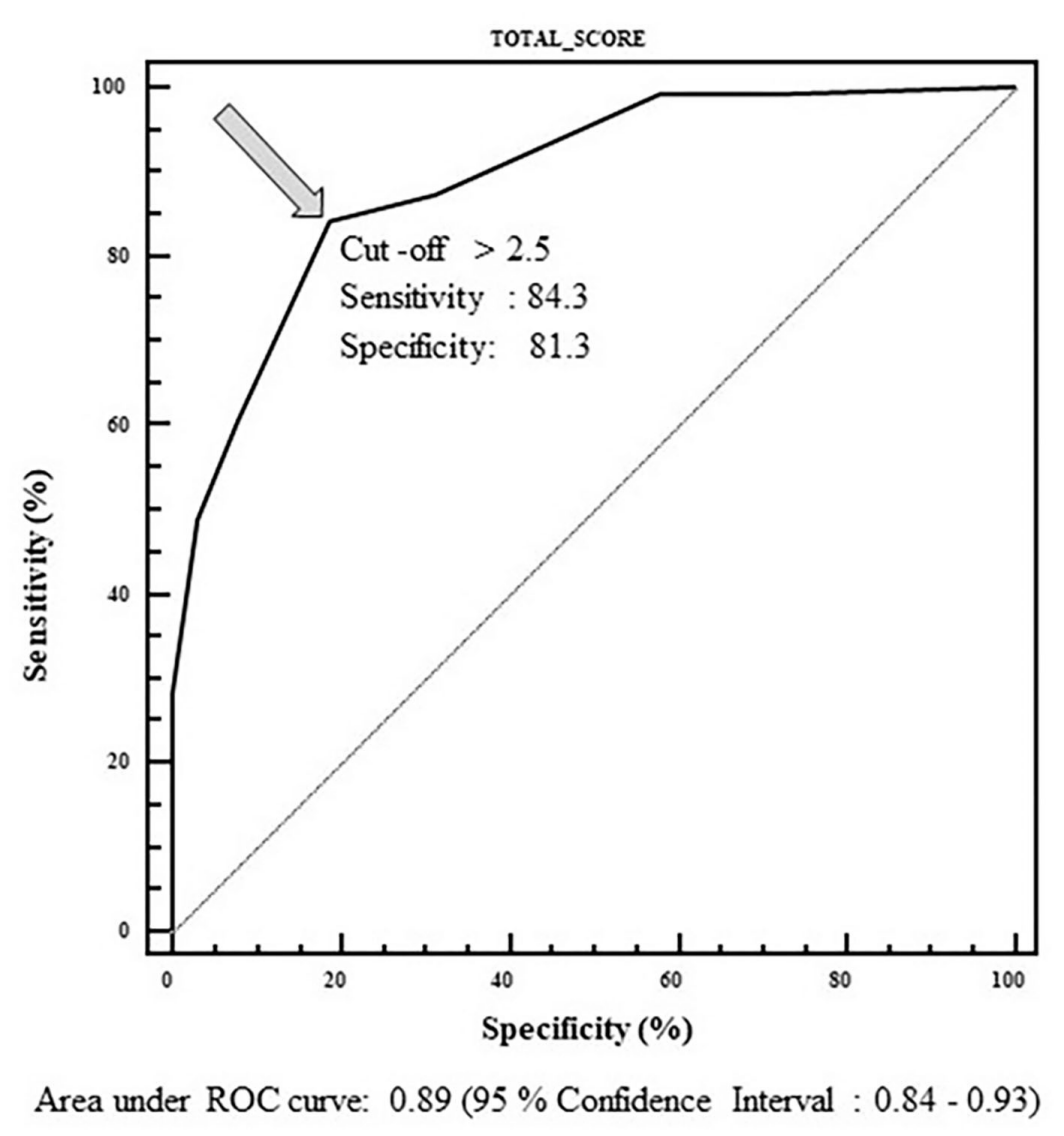
ROC curve used to determine cut-off value of the total score able to predict surgical intervention. Range of the score: 0–8.5. Best cut-off value: 2.5.

For each kidney, the total score (that theoretically ranges between 0 and 8.5) was then calculated; the best cut-off score was calculated by means of an ROC curve, using the surgical intervention as the outcome variable (yes/no). The best cut-off score able to predict surgical intervention was > 2.5 (Figure 1).

Outcome data at the end of the follow-up was available on 148/200 kidneys (74%). The median length of follow-up was 1.73 years (1st−3rd q: 0.90–3.40 years) in the surgical group, and 1.69 years (1st−3rd q: 0.91–4.49 years) in the non-surgical group.

As shown in Table 4, a greater percentage of improvement was observed in operated kidneys (75 out of 104, 72.1%) with respect to non-operated kidneys (15 out of 44, 34.1%); this difference was highly statistically significant (Fisher's exact test, *P* < 0.0001). Moreover, analysis of the observed outcome at the end of follow-up showed that diagnosis of UPJ Intrinsic Obstruction (UPJ-IO) is the subtype that had the greatest improvement after surgery (Fisher's exact test, *P* < 0.001); similar data were observed for the diagnosis of Intrinsic Cicatricial UPJ (UPJ-IC) and Extrinsic Vascular UPJ (UPJ-EV) without reaching statistical significance due to the small numbers in the two diagnostic subgroups. Data of the outcome at last follow-up was available for only one kidney with the diagnosis of Extrinsic Adherential UPJ (UPJ-EA) and therefore no statistical test could be performed for this diagnostic subgroup; it was only observed that this case had improved.

**Table 4:** Observed outcome at the end of follow-up (*N* = 148/200).

	**Improved**	**Stable**	**Deteriorated**	***P***
	***N* (%)**	***N* (%)**	***N* (%)**	
Operated kidney [*n* = 104]	75 (72.1%)	26 (25.0%)	3 (2.9%)	0.0001^§^
Non-operated kidney [*n* = 44]	15 (34.1%)	25 (56.8%)	4 (9.1%)	
**Diagnosis at MRI:**				
**UPJ-IO [*****n*** **=** **75]**				
Operated kidney [*n* = 59]	45 (76.3%)	13 (22.0%)	1 (1.7%)	0.001^§^
Non-operated kidney [n=36]	14 (38.9%)	19 (52.8%)	3 (8.3%)	
**UPJ-IC [*****n*** **=** **15]**				
Operated kidney [*n* = 9]	5 (55.6%)	4 (44.4%)	-	
Non-operated kidney [*n* = 6]	1 (16.7%)	5 (83.3%)	-	0.29^§^
**UPJ-EV [*****n*** **=** **37]**				
Operated kidney [*n* = 35]	24 (68.6%)	9 (25.7%)	2 (5.7%)	0.05^§^
Non operated kidney [*n* = 2]	-	1 (50.0%)	1 (50.0%)	
**UPJ-EA [*****n*** **=** **1]**				
Operated kidney [*n* = 1]	1 (100.0)	-	-	-
Rene non-operato	-	-	-	

*Figures in round parentheses represent row percentages*.

*^§^P: Fisher's exact test*.

## Discussion

The diagnosis of congenital hydronephrosis has increased in incidence since the 1980s due to the greater diffusion of prenatal US. In published literature, about 50–75% of hydronephrosis are resolved or stabilized without treatment; the remaining cases need corrective surgery as chronic dilation of the calico-pyelic system induced by permanent obstacle to urinary outflow determines an incomplete morpho-functional development of the affected kidney (2, 4, 5, 24, 25).

Research has shown that early diagnosis of hydronephrosis improves the overall management in the postnatal phase (6, 26). No univocal consensus on management exists. The above-mentioned surgical indications for corrective surgery of the UPJO are limited as they are based on the need for an existing functional renal damage (8, 22, 27, 28).

The most frequent cause of UPJO (62–75%) is the presence of an aperistaltic segment adjacent to the pyelo-ureteral junction. Initially, the obstruction is only functional, however, with time tissue alterations can occur, causing scarring and reduction of the pyelo-ureteral caliber (29–32). The second most frequent cause of UPJO is the presence of an abnormal vessel (11–49%) that crosses anteriorly the UPJ or proximal ureter, hindering the outflow (31, 33–35). According to literature we found that 48/192 patients (24%) had obstructive CV. In these cases, the obstruction may be intermittent, with colic symptoms and later diagnosis. Detection of CV by imaging studies as a cause of obstruction is important as it may represent a surgical indication for the risk of ischemia due to the compression of the CV by the dilated pelvis (36). Moreover, we found that patients with obstructive CV had a median APD pre-Furosemide of 26.5 mm (17–35.8 IC) which is greater than the cut off value for surgery identified by our statistical analyses.

**Figure 2:**
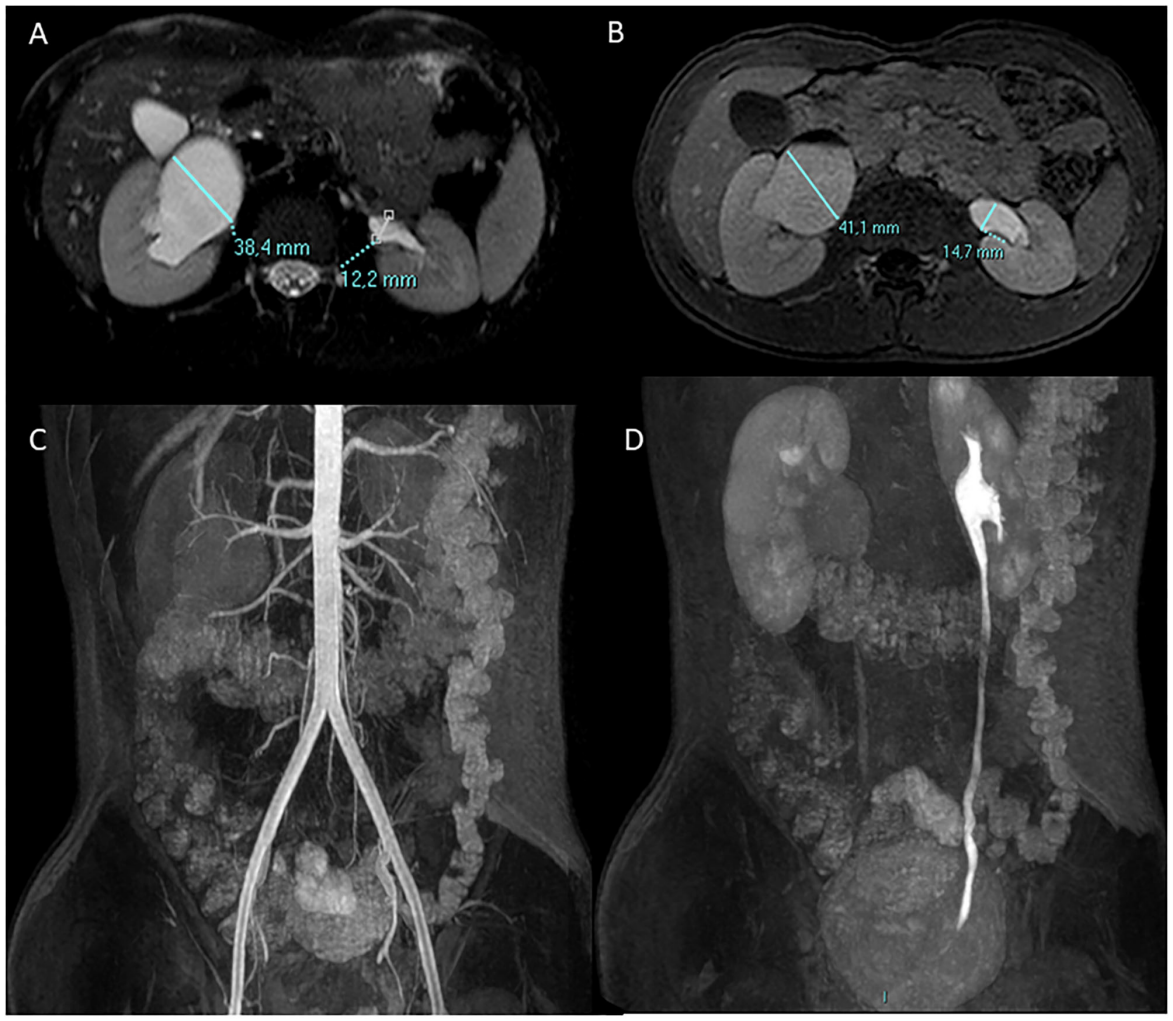
A 13-year-old boy with right UPJO due to a crossing lower pole accessory renal artery. Antero-posterior diameter of the pelvis is measured in the axial plane pre **(A)** and post **(B)** Furosemide injection. Coronal angiographic gradient echo 3D sequence post contrast medium injection **(C)** shows the right lower pole accessory renal artery which crosses the homolateral UPJO, the urographic phase is delayed in comparison with the contralateral side as shown in the coronal 3D gradient echo urographic sequence **(D)**.

Identification of the abnormal vessel in preoperative imaging remains an important step in UPJO surgical planning (Figure 2) avoiding unnecessary pyeloplasty (31, 37). Menon et al. identified radiological findings on MRU suspicious for the presence of CV (small, intrarenal, globular pelvis, widening of the pyelo-calyceal junction, and prominent calyceal dilation) (38). Fiorenza et al. found that fMRU was helpful to identify CV in 89.2% of cases (39).

DRS is the gold standard for the functional evaluation of hydroureteronephrosis and to guide therapeutic decisions; fMRU provides a radiation-free morphological and functional assessment with remarkably high spatial and contrast resolution (33, 40). Recently, published data demonstrated the good concordance between fMRU and DRS findings in patients with CAKUT (18).

Furthermore, in recent years, several studies have been published on the development of imaging-based scores for the assessment of hydroureteronephrosis, able to objectively predict the need for surgery and to evaluate the resolution of the obstruction (19, 41).

In this study, we focused on the role of fMRU in guiding surgical or conservative approach in UPJO proposing a quantitative score. The score was obtained using the classical statistical methodology but also taking into account clinical considerations.

First, variables considered as items of the score were chosen on a clinical basis. Subsequently, the considered variables were evaluated according to a standard statistical method with a multi-variable logistic regression analysis, also being used to identify the best predictors of surgical interventions. The beta coefficients of the best-fitted logistic model were considered as points of the score for each significant variable. Finally, all these scores were then added up to obtain a “total prediction score” for each individual patient.

In our bivariate model analysis between surgical and non-surgical kidneys, the presence of an abnormal vessel obstructing the UPJ (93.8%) showed to be the morphological parameter with the greatest statistical significance. Other parameters, such as morphology and rotation of the pelvis and ureteral dilation were not found to be statistically significant when comparing the two groups. Interestingly, our analysis highlighted the role of dilation of the calyxes and renal pelvis measured both pre and post-hydration and Furosemide injection. Surgical kidneys had a considerably greater dilation than non-surgical ones, which worsened after water load and Furosemide injection.

The subclass of extrinsic juntopathy resulted to be the major condition candidate to surgery.

A substantial concordance was found between surgical findings and fMRU, consistent with published data (38, 42).

At follow-up, the hydronephrosis and the morphology of the pathological kidney were evaluated as improved, stable, or worsened. An important finding of our analyses is that surgical kidneys improved significantly in 88.1% of cases, while non-surgical kidneys improved in 52% of cases or remained stable in 44% of cases. Intrinsic UPJOs have the most statistically significant improvement after treatment. Moreover, UPJ-EV is always treated surgically with an improvement of the hydronephrotic condition.

Based on the regression model, the significant predictive variables were pelvic dilation, fMRU diagnosis, and the urographic phase. Functional curves, significant at the bivariate analysis, were excluded from the final score because of their direct correlation with other variables.

The scores attributed to each predictive value are shown in Table 3. The score assigned to the delayed and incomplete phase is higher than the absent one, probably due to the few cases present in the latter group. The discrepancy between the severity of the urographic framework and the numerical value of the score is certainly to be attributed to the sample selection; we excluded all cases of severe UPJO associated with other congenital anomalies that often require surgery. The parameter AUC values greater or lower than 38, even if significant at bivariate analysis, was excluded from the score because it resulted to be not significant at the multivariate analysis. However, its role in surgical planning remains a corner stone.

The more delayed the detection of obstructive disease, the more likely the patient will undergo follow-up instead of surgery; conversely, very young patients have a higher risk of undergoing surgery.

The best cut-off value obtained by the ROC curve method, able to discriminate between subjects who had to be operated or not, was calculated. The ROC curve demonstrates the reliability of our score in discriminating surgical/non-surgical patients with a cut-off >2.5, a sensitivity of 84.3%, and specificity of 81.3%.

However, our study has some limitations. Within the surgical kidney group, we had few patients with UPJ-EA. Furthermore, we had fewer cases with available follow-up data in non-surgical kidneys; however, the median of the follow-up of the non-surgical and surgical groups, is substantially overlapping (1.7 and 1.3 years) and represents an adequate period to express clinical judgments on the pathological kidney (improved, stable, worsened).

Finally, this new radiological score still needs to be applied to a wider different population to be validated.

In conclusion, by means of a bivariate and multivariate analysis, we evaluated the predictive role of fMRU findings in the discrimination between surgical and non-surgical patients.

Based on our experience we propose a new highly sensitive and specific fMRU score able to identify surgical vs. non-surgical kidneys in patients with UPJO.

We believe that this score can help surgeons in patients' management, however it needs to be clinically validated in a large cohort of patients. Hopefully, a wider use of this score will further improve its clinical use. Considering that the main goal of pediatric urology is to preserve long term renal function with the least invasive approach, we strongly believe that this tool may be useful in guiding clinical decisions.

## Data Availability Statement

The raw data supporting the conclusions of this article will be made available by the authors, without undue reservation.

## Ethics Statement

Ethical review and approval was not required for the study on human participants in accordance with the local legislation and institutional requirements. Written informed consent from the participants' legal guardian/next of kin was not required to participate in this study in accordance with the national legislation and the institutional requirements.

## Author Contributions

MBD and FS wrote the original draft of the paper. AP performed statistical analyses. MCYW, IC, MC, LB, LA, MB, GP, GMG, and GM made contributions. All authors approved the final version of the paper.

## Funding

This work was supported by ministerial funds (MSALRF DEL68/21) as it is part of the project precision medicine in CAKUT.

## Conflict of Interest

The authors declare that the research was conducted in the absence of any commercial or financial relationships that could be construed as a potential conflict of interest.

## Publisher's Note

All claims expressed in this article are solely those of the authors and do not necessarily represent those of their affiliated organizations, or those of the publisher, the editors and the reviewers. Any product that may be evaluated in this article, or claim that may be made by its manufacturer, is not guaranteed or endorsed by the publisher.
